# Ultrafast Coherent Exciton Couplings and Many-Body Interactions in Monolayer WS_2_

**DOI:** 10.1021/acs.nanolett.4c01991

**Published:** 2024-06-20

**Authors:** Daniel Timmer, Moritz Gittinger, Thomas Quenzel, Alisson R. Cadore, Barbara L. T. Rosa, Wenshan Li, Giancarlo Soavi, Daniel C. Lünemann, Sven Stephan, Martin Silies, Tommy Schulz, Alexander Steinhoff, Frank Jahnke, Giulio Cerullo, Andrea C. Ferrari, Antonietta De Sio, Christoph Lienau

**Affiliations:** † Institut für Physik, Carl von Ossietzky Universität Oldenburg, 26129 Oldenburg, Germany; ‡ Cambridge Graphene Centre, University of Cambridge, CB3 0FA Cambridge, United Kingdom; § Institute for Theoretical Physics and Bremen Center for Computational Materials Science, University of Bremen, P.O. Box 330 440, 28334 Bremen, Germany; ∥ Dipartimento di Fisica, Politecnico di Milano, Piazza L. da Vinci 32, 20133 Milano, Italy; ⊥ Istituto di Fotonica e Nanotecnologie-CNR, Piazza L. da Vinci 32, 20133 Milano, Italy; ■ Center for Nanoscale Dynamics (CENAD), Carl von Ossietzky Universität Oldenburg, Institut für Physik, 26129 Oldenburg, Germany

**Keywords:** transition metal dichalcogenides, ultrafast spectroscopy, many-body interactions, coherent couplings

## Abstract

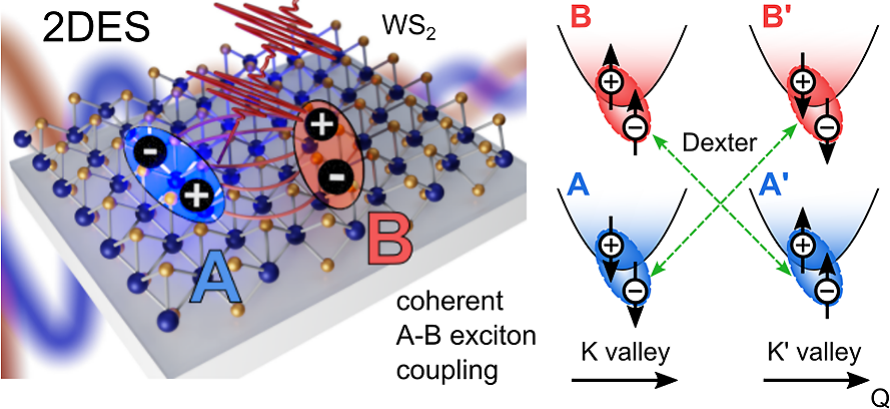

Transition metal dichalcogenides (TMDs) are quantum confined systems with interesting optoelectronic properties, governed by Coulomb interactions in the monolayer (1L) limit, where strongly bound excitons provide a sensitive probe for many-body interactions. Here, we use two-dimensional electronic spectroscopy (2DES) to investigate many-body interactions and their dynamics in 1L-WS_2_ at room temperature and with sub-10 fs time resolution. Our data reveal coherent interactions between the strongly detuned A and B exciton states in 1L-WS_2_. Pronounced ultrafast oscillations of the transient optical response of the B exciton are the signature of a coherent 50 meV coupling and coherent population oscillations between the two exciton states. Supported by microscopic semiconductor Bloch equation simulations, these coherent dynamics are rationalized in terms of Dexter-like interactions. Our work sheds light on the role of coherent exciton couplings and many-body interactions in the ultrafast temporal evolution of spin and valley states in TMDs.

1L-TMDs are quantum materials^1^ with a wide range of applications thanks to their unusual optoelectronic properties.^2−4^ The quantum confinement and the reduced dielectric screening allows for strongly bound excitons^5,6^ with large binding energies of several 100 meV,^2^ excited Rydberg states,^6^ highly correlated multiparticle excitations^7−10^ and strong many-body interactions.^5,11^ Their large optical transition dipole moment^12^ and sensitivity to the dielectric environment of the excitons makes TMDs ideally suited to tailor the optical properties via their surrounding,^13,14^ or by utilizing strong coupling in microcavities^15,16^ and plasmonics.^17,18^ Strong spin–orbit coupling of the direct bandgap 1L-TMDs gives rise to energetically well-separated A and B excitons.^2^ Their spin-valley locking can be exploited for selective excitation of the K and K’ valleys of the hexagonal Brillouin zone (Figure 1a) by using circularly polarized light.^2^ When using linearly polarized light, both excitons in both valleys can be excited.

**Figure 1:**
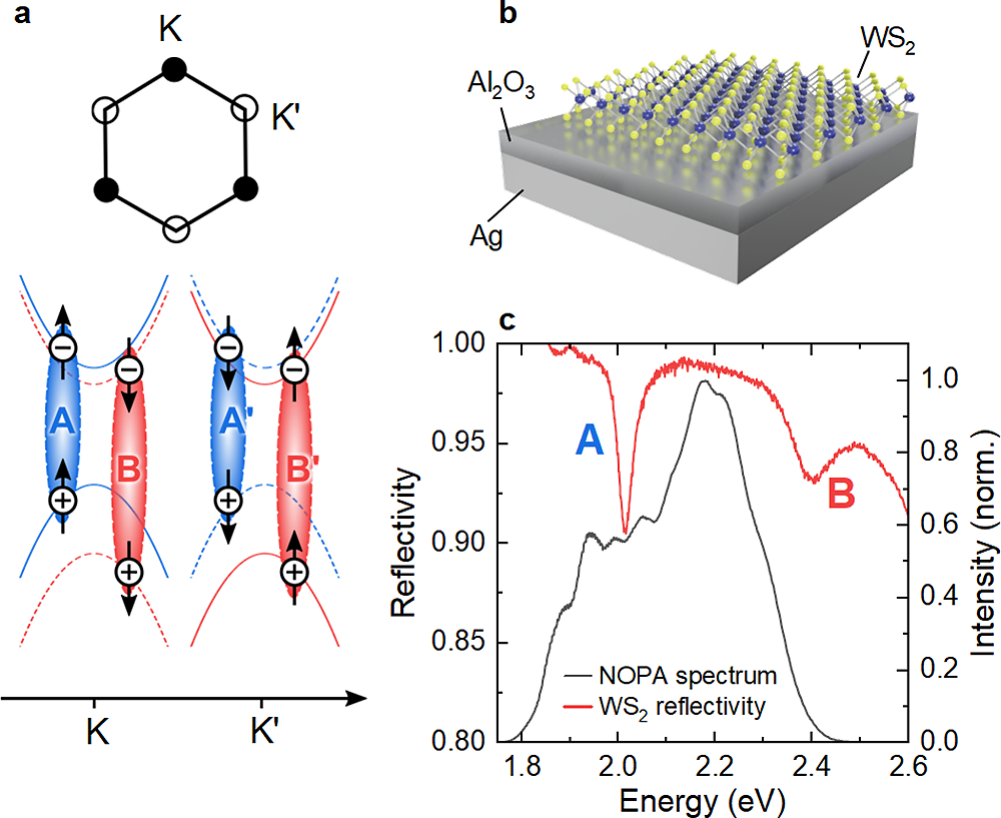
(a) Schematic momentum-space representation of the hexagonal unit cell with K and K’ valleys. Direct excitons are depicted as Coulomb-bound e–h pairs with their spins indicated by arrows. When using linearly polarized light, all excitons can be excited. (b) A 1L-WS_2_ flake is deposited on a Ag substrate coated with 5 nm Al_2_O_3_. (c) Reflectivity (red) showing the A and B excitons at ∼2.01 and ∼2.4 eV, respectively. The laser spectrum (black) is tuned to cover both resonances, while predominantly exciting the A exciton.

These properties make TMDs promising for valleytronics,^19−21^ exploiting differences in the valley population as information carrier.^19−21^ However, intra- and intervalley Coulomb correlations among excitons in TMDs lead to finite, (sub-) ps lifetimes of the valley polarization.^22−24^ Both theoretical^24−28^ and experimental^22−26,29−31^ studies demonstrated that a simplistic picture where excitons are treated as independent does not hold in these materials, and both inter- and intravalley coupling mechanisms lead to a mixing of the excitonic states. A Dexter-like interaction that directly couples excitons with the same spin in different valleys was suggested,^25,27,30^ and a coupling strength in the range of 50 meV was estimated for 1L-WS_2_.^25^ This mechanism can be understood as a near-field dipole–dipole coupling between an optically induced exciton polarization in one valley and a polarization in the other valley, leading to a coherent population transfer.^25^

Two-dimensional electronic spectroscopy (2DES)^32−34^ is an ideal tool to study these many-body interactions and coherent exciton couplings because it provides direct signatures of such couplings in the form of cross peaks in the 2DES maps. In this extension of ultrafast pump–probe spectroscopy,^35^ a pair of phase-locked pump pulses is used to record energy–energy maps as a function of the pump–probe delay,^35,36^ correlating the excitation and detection energies of the optical nonlinearities of the investigated material. One essential characteristic of this technique is that correlations between energetically separate states due to coherent or incoherent couplings manifest as cross-peaks in a 2DES map.^32,35,36^ These and their dynamics can give deep insight into the quantum dynamics of the system upon photoexcitation.^32,36^ 2DES has been used to study many-body interactions and couplings in 1L-TMDs,^23,29,30,37,38^ their heterostructures^39−41^ and microcavities.^42^ Refs (29 and 30) assigned cross-peaks between A and B excitons in 1L-MoS_2_ as direct intravalley and intervalley Dexter-like interactions, respectively. Ref (29) reported a coupling strength of 28 meV in 1L-MoS_2_, therefore a weak mixing between the A and B exciton states on the order of a few percent.^29^ Despite their significant energetic detuning, such a coherent coupling should give rise to coherent population oscillations between the two states with an oscillation period inversely proportional to the detuning.^36^ Such oscillations should be observable in time-resolved experiments probing the exciton populations. Weak mixings^29^ and short oscillation periods in the 10 fs range, governed by the detuning between A and B excitons,^27^ make such experiments challenging.

Here, we report ultrafast and broadband pump–probe and 2DES measurements on 1L-WS_2_, demonstrating many-body interactions and coherent couplings between A and B excitons at room temperature (RT). We observe 11.5 fs oscillations of the B exciton resonance in pump–probe and cross-peaks between the A and B excitons in the 2DES maps as a signature of coherent exciton coupling. We deduce a coupling strength of ∼50 meV. Microscopic simulations based on the semiconductor Bloch equations (SBE)^43^ suggest that these oscillations mainly reflect Dexter-like intervalley interactions. The deduced coupling strength is in agreement with earlier estimates.^25,27^ The coherent interactions, and therefore the exciton response, may be tailored by strongly coupling the 1L-TMD to vacuum field fluctuations of external cavities.

To explore exciton couplings in 1L-TMDs, we prepare 1L-WS_2_ flakes by micromechanical exfoliation of bulk 2H-WS_2_. Selected flakes are stamped onto a polycrystalline Ag film with a thickness of 200 nm, coated with a 5 nm aluminum oxide layer to avoid hot electron transfer between Ag and TMD.^44−46^ The samples are characterized by Raman and photoluminescence spectroscopy^47^ (see Figure S3 of the Supporting Information, SI).

Figure 1b plots the RT sample reflectivity (red curve), showing two resonances at 2.01 and 2.4 eV, consistent with the 1s states of A and B excitons,^48^ respectively. The incident light is reflected by the Ag mirror, thus passes twice through the 1L-WS_2_. Hence, the experiment essentially probes the sample transmission and therefore the imaginary part of the susceptibility.^49,50^ The increased full width at half maximum of the B (122 meV) compared to the A exciton (33 meV) resonance was assigned to an intervalley scattering from B to A,^26^ and Dexter-like coupling of the B exciton to a continuum of A exciton states in the opposite valley.^26^ The reflectivity also shows a broad background associated with higher-energy interband transitions at K and K’,^12^ but may also include transitions at the Γ points.^5,12^

To investigate the interactions between A and B excitons in the regime of coherent valley polarizations,^30^ we perform ultrafast pump–probe and 2DES experiments utilizing pulses generated using a noncollinear optical parametric amplifier (NOPA) pumped with a 175-kHz fiber amplifier system^36,50^ (see SI, Section 1). The NOPA delivers 9 fs pulses (Figure S2) with a spectrum presented in Figure 1b (black curve), tuned to overlap both exciton resonances. The laser spectrum is tailored to cover the B exciton, while minimizing the excitation of excitons and free carriers with energies above the B exciton. The pump–probe and 2DES setup^36,50^ utilizes an in-line common-path interferometer based on birefringent wedges^51,52^ to generate a collinear, phase-stable pulse pair for 2DES. In the pump–probe geometry, differential reflectivity spectra Δ*R*/R = (*S*_*on*_–*S*_*off*_)/*S*_*off*_ are obtained by recording the reflected probe beam in the presence (*S*_*on*_) and absence (*S*_*off*_) of the pump,^36,50^ using a fast line camera with 87.5 kHz readout rate and high-frequency mechanical chopping at half the readout rate. A sequence of Δ*R*/*R* spectra is recorded by scanning the pump-pulse delay at a fixed waiting time *T* between the second pump pulse and probe. The Fourier transform of the Δ*R*/*R* spectra along the delay axis gives the 2DES map at *T*.^36,51,52^ For pump–probe measurements, both pump pulses coincide, see SI for more details. All experiments are performed at RT, in reflection geometry, with colinearly polarized pump and probe pulses. We use a sufficiently weak^53^ excitation density of ∼1.7 · 10^12^ cm^–2^ (see SI Section 3), well below the Mott transition of 1L-WS_2_^54^ and within the regime of perturbative third-order nonlinearities^55^ (Figure S4).

Pump–probe measurements recorded for *T* up to 1.5 ps on 1L-WS_2_ are presented in Figure 2. The pump–probe map in Figure 2a shows two distinct positive resonances at the energies of the A and B excitons. For positive waiting times, a weaker negative background signal is seen for all other detection energies. Spectral crosscuts (Figure 2b) allow for more insights into the sample nonlinearity. Since we are probing the imaginary part of the sample susceptibility, positive signals are usually associated with ground state bleaching^35,55^ or stimulated emission signals,^35,55^ while negative ones indicate excited state absorption.^35,55^

**Figure 2:**
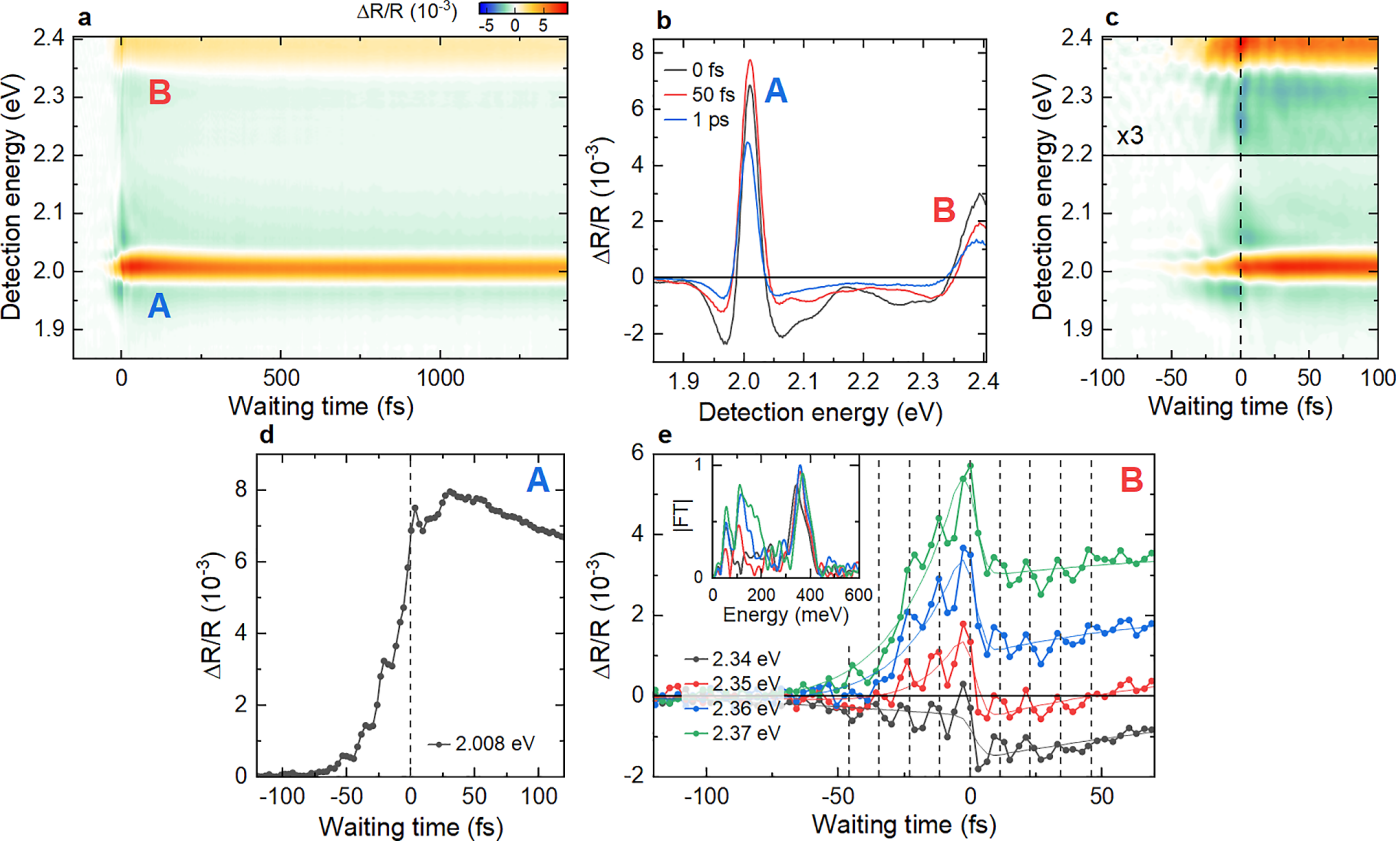
Ultrafast pump–probe spectroscopy of 1L-WS_2_ on a Ag substrate. (a) Pump–probe map of ΔR/R showing positive nonlinearities (ΔR/R > 0) at the A and B exciton energies. (b) Spectral crosscuts at selected waiting times showing the characteristic line shape of an EID nonlinearity, most clearly for the A exciton. (c) Zoom-in at early waiting times. The B exciton resonance shows pronounced oscillations at positive and negative *T*. (d) Waiting time dynamics of the A exciton resonance. For *T* < 0, the PPFID of the A exciton can be seen. For *T* > 0, the dynamics show an initial spike and delayed rise of the A exciton signal. (e) Dynamics at selected energies for the B exciton resonance showing pronounced ΔR/R oscillations with a period ∼11.5 fs (dashed vertical lines). A Fourier transform of the oscillatory component confirms an energy of ∼360 meV, in good agreement with the A–B exciton splitting. These oscillations are the signature of coherent coupling between A and B excitons.

This picture, usually applied in atomic^32^ and molecular^35,56^ systems, cannot fully account for the optical nonlinearities of electronically correlated systems such as semiconductor excitons.^11,32,33^ For these excitonic systems, many-body interactions such as excitation-induced shifts (EIS)^11,57^ and dephasing (EID)^11,38,58^ dominate the nonlinear response. Indeed, the A exciton resonance in Figure 2b shows the typical EID line shape^58,59^ of a narrow positive peak superimposed on a broader negative resonance (Figure S6). The line shape results from a broadening of the exciton resonance after excitation by the pump. This characteristic EID line shape agrees well with that reported in the pump–probe measurements of ref (11) for selective narrowband excitation of either A or B excitons. The broadening of A excitons increases with pump excitation density, as can be seen in a study of fluence dependent spectra in Figure S4, characteristic for TMDs.^11,38^ Similar lineshapes are also seen for the B exciton.

We now discuss the dynamics of the spectra in Figure 2c during the first 100 fs. At negative times, the sample shows the characteristic signatures of a pump-perturbed free induction decay (PPFID)^59,60^ of the exciton resonances. The PPFID can also be seen in a slow buildup of the differential reflectivity for *T* < 0 of the A exciton in Figure 2d. In addition, after some initial sharp spike at *T* = 0, we observe a delayed rise in the signal within ∼30 fs, followed by biexponential decay with time constants of ∼150 fs and 10 ps. For the B exciton resonance, close inspection in Figure 2c reveals oscillatory modulations around *T* = 0. Traces at selected detection energies in Figure 2e show these oscillations with a period of ∼11.5 fs more clearly. Since our pulse duration is only slightly shorter than the oscillation period, even larger oscillation amplitudes are expected when further improving the time resolution of the experiment. Fourier transforms of the oscillatory component (inset) show an associated energy of ∼360 meV, close to the splitting between A and B excitons of ∼380 meV deduced from Figure 2b. No additional long-lived oscillations reflecting, e.g. coherent phonons, are observed in our waiting time window.

To study the origin of these high-frequency oscillations, we now turn to 2DES measurements. 2DES maps at selected waiting times (Figure 3a-d) show, in detail, signatures of many-body interactions and coherent couplings between A and B excitons. In addition to the (A,A) and (B,B) diagonal peaks, the maps show (A,B) and (B,A) cross-peaks. We denote with (ex,det) the resonance along the excitation (ex) and detection (det) axis. All cross-peaks are already present at *T* = 0 fs. Such cross-peaks have also been observed for 1L-MoS_2_ in refs (30 and 29) and indicate couplings between A and B excitons. In our experiments, the cross-peaks are partially covered by additional and prominent vertical stripes along the excitation axis at energies above the A exciton. Such vertical stripes are taken as a signature of many-body interactions, specifically EID, between an exciton and a higher-lying continuum of states.^61,62^ For 1L-WS_2_, the quasiparticle bandgap is expected close to the B exciton resonance,^6,53^ although the exact energy can be strongly affected by the dielectric environment.^13^ The vertical stripes in Figure 3 may arise from free carrier excitations at the K and K’ valleys, but can also be caused by the excitation of higher-energy Coulomb-correlated states. Excitation of such states is already indicated by the linear reflectivity spectrum.

**Figure 3:**
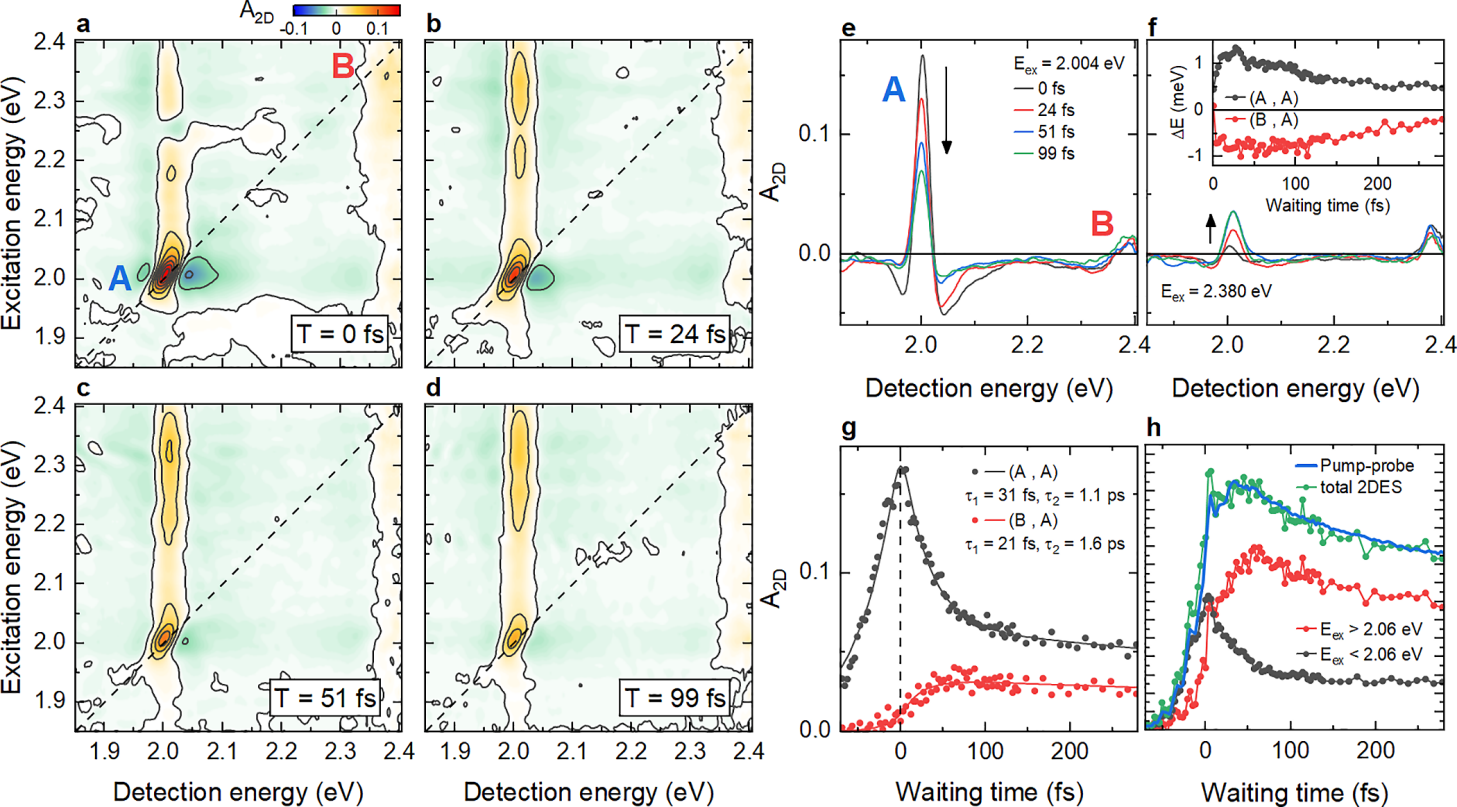
2DES of 1L-WS_2_. (a–d) 2DES maps at selected *T*. In addition to diagonal features at the A and B exciton energy, (A,B) and (B,A) cross-peaks arise from a coherent coupling between the two types of excitons. Vertical stripes for excitation energies above the A exciton at both the A and B exciton detection energy are signatures of many-body effects, such as EID, by an additional broad background density of states. (e,f) Evolution of spectral crosscuts at the A (e) and B (f) exciton excitation energy. The A exciton resonance undergoes a reduction in amplitude and change in symmetry within ∼50 fs, reflecting an EIS of the resonance energy by Δ*E* (inset in f). (g) Dynamics of the diagonal (A,A) and cross (B,A) peak. Within the first ∼50 fs, the (A,A) exciton amplitude partially decays, while the (B,A) cross-peak amplitude builds up. (h) Spectrally integrated 2DES trace, detected at the A exciton (green dots), together with pump–probe dynamics (blue line) of the A exciton. Similar 2DES traces, now integrated over excitation energies larger than (red) or smaller than (black) 2.06 eV. A delayed rise in ΔR/R reflects the relaxation of B exciton populations into the A exciton on a sub-50 fs time scale.

We now focus on the A exciton resonance in the 2DES maps. The (A,A) peak shows a finite tilt toward the diagonal, a sign for a finite inhomogeneous broadening that may be caused by local strain^40^ or disorder.^38^ The resonance also shows pronounced negative EID wings with a line shape that changes substantially during the first ∼50 fs. Spectral crosscuts at the A exciton excitation energy in Figure 3e indicate that the line shape becomes strongly asymmetric within 25 fs (black to red line) before it evolves back into a more symmetric one at later *T*. The initial change in line shape is accompanied by a red-shift of the peak maximum by ∼2.5 meV (black to red line). In contrast, a rapid blue-shift of the (B,A) cross-peak maximum is seen in Figure 3f. Such a change in resonance energy suggests that not only EID, but also EIS, is affecting the Δ*R*/*R* lineshapes. Very similar lineshapes^11^ were reported in a pump–probe study of 1L-WS_2_ for selective excitation of A or B excitons with 100 fs pulses.

To more quantitatively analyze the shift dynamics, spectral crosscuts of the differential reflectivity are fitted by the difference between two Lorentzians. Many-body effects induced by optical pumping are phenomenologically included by a fixed broadening (EID) and an energy shift Δ*E* (EIS) of the resonances when the pump is switched on. The inset of Figure 3f depicts Δ*E* of the exciton resonance as deduced from the analysis of the (A,A) peak (black) and (B,A) peak (red), respectively. Δ*E* is opposite in sign to the spectral shift seen in Δ*R*/*R* (Figure, 3e) since such a pump-induced shift results in an asymmetric line shape with a maximum turned into the opposite direction. For (A,A), we find a rapid blue-shift within ∼25 fs that decays on a longer, 200 fs time scale. The analysis of the (B,A) peak indicates a red-shift of the A exciton by the pump, matching the observed blue-shifts in the cross cuts of Figure 3f. The Δ*E* dynamics deduced from (B,A) are similar to those from (A,A). In ref (11), qualitatively similar energetic shifts of the A exciton, also with opposite sign for A and B excitation, were rationalized by a complex interplay of intra- and intervalley Coulomb interactions between the excitons.

We now turn to the amplitudes of the (A,A) and (B,A) peaks in Figures 3e-f. These display a rapid decay of the diagonal feature, while the cross-peak amplitude builds up. A fit to the peak dynamics in Figure 3g yields time constants of ∼31 fs for the initial (A,A) decay and a rise-time of ∼21 fs for the cross-peak, a similar time scale as observed for the initial energy shifts in Figure 3f (inset). Since the pump–probe spectra can be retrieved by integrating the 2DES signal along the excitation energy axis (projection slice theorem^55^), we can now identify the origin of the delayed rise of the pump–probe data (Figure 3h, blue). It reflects the competition between fast decay and rise in the excitation-energy dependent 2DES (Figure 3h, red and black). This rise in signal may reflect an incoherent relaxation of B excitons or other high-energy excitations into the A exciton. Earlier measurements of an exciton formation time from free carriers of 30 fs in 1L-MoS_2_ would support this assignment.^49,63^ More complex many-body interactions may also describe the spectral evolution.^27^

In order to provide a more in-depth analysis of our data and to give an estimate of the coherent A-B coupling strength, we perform nonperturbative density matrix simulations of the pump–probe and 2DES data using the Lindblad master equation^36,64^ (see SI Section 5). In these calculations, we consider a minimal Hamiltonian (Figure 4a), comprising one A-exciton |*X*_*A*_⟩ and one B-exciton state |*X*_*B*_⟩, while neglecting the valley degree of freedom of the excitons. The Hamiltonian introduces three two-quantum (2Q) states, |*X*_*A*_*X*_*A*_⟩, |*X*_*B*_*X*_*B*_⟩ and the mixed state |*X*_*A*_*X*_*B*_⟩. The addition of 2Q states to the one-quantum (1Q) states allows us to phenomenologically account for many-body effects, specifically EID and EIS, in the exciton manifold.^32,34^ Here, we only introduce a finite amount of EID, since we expect that this dominates the nonlinearity. For this, we increase the dephasing rates of the |*X*_*A*_*X*_*A*_⟩ and |*X*_*B*_*X*_*B*_⟩ states within the Lindblad formalism by 10% relative to those of the corresponding 1Q states. This introduces a nonlinearity by increasing the line width of the 1Q → 2Q transitions relative to that of the 0 → 1Q transitions, effectively breaking the symmetry of the system. No EID effects are introduced for the mixed |*X*_*A*_*X*_*B*_⟩ state. The results of these simulations are summarized in Figure 4.

**Figure 4:**
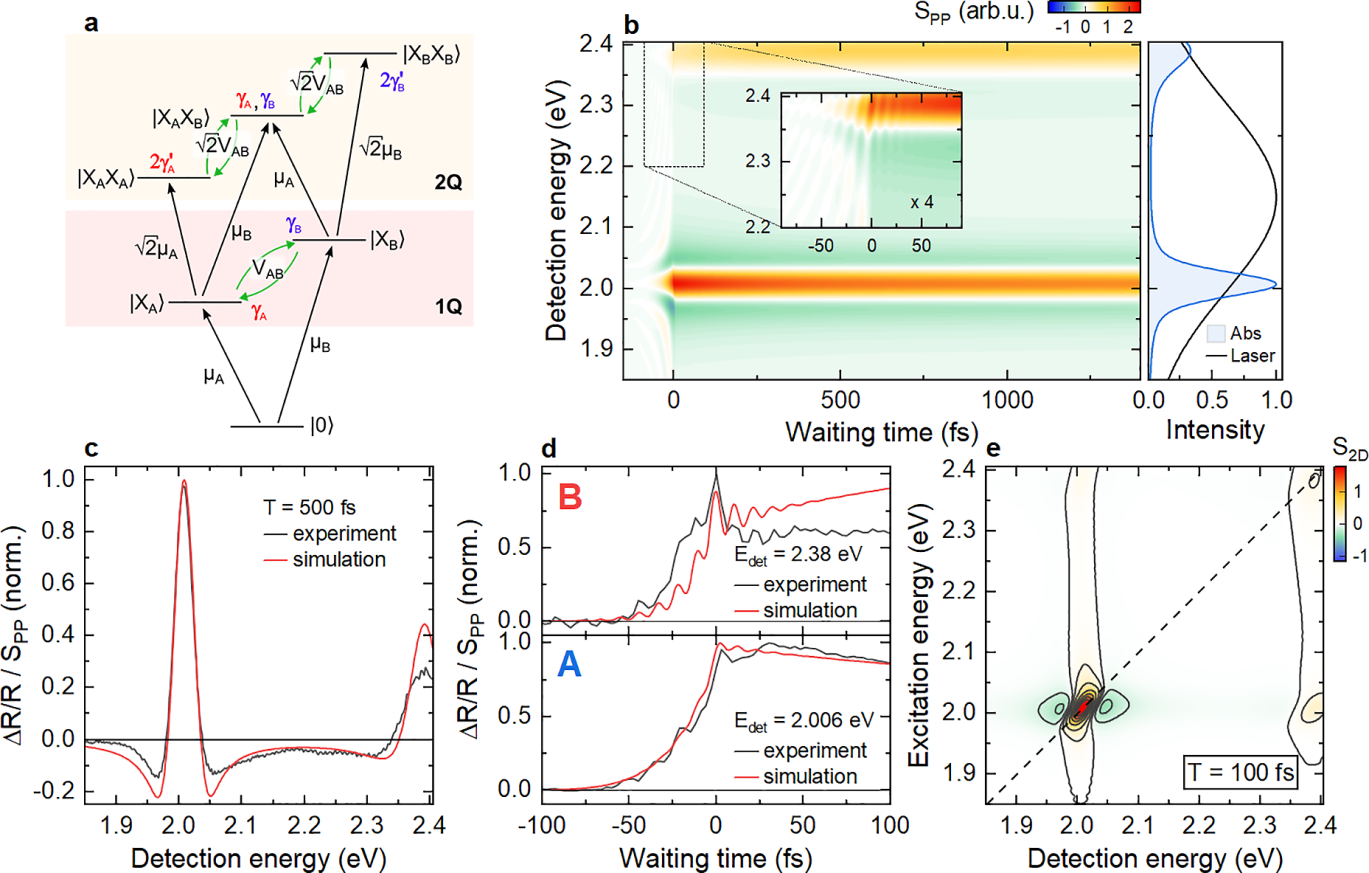
Simulation of ΔR/R measurements considering a 50 meV coupling between A and B excitons. (a) Scheme of the employed many-body Hamiltonian. In addition to the 1-quantum (1Q) states |X_A_⟩ and |X_B_⟩, three two-quantum (2Q) states are considered to phenomenologically account for many-body interactions. Different types of system–bath interactions with a dephasing rate γ are indicated. EID is introduced by altering the dephasing rates associated with the 2Q states. (b) Simulated pump–probe map (left). The absorption (blue) and laser spectra (black) are shown in the right panel. The simulations reproduce the exciton nonlinearities observed experimentally. The inset highlights the rapid ΔR/R oscillations induced by the A–B coupling. (c) Comparison between experimental (black) and simulated (red) ΔR/R line shape showing the dominant EID nonlinearity. (d) Experimental (black) and simulated (red) ΔR/R dynamics at the A and B excitons. The rapid oscillations at early times are qualitatively reproduced. (e) Simulated 2DES map at *T* = 100 fs, showing the dominant resonant EID nonlinearity of the diagonal peaks and cross peaks arising from the coherent A–B coupling.

The simulations reproduce both the amplitudes of experimental 2DES cross-peaks, as well as the amplitude and period of the coherent population oscillations in Figure 2, reasonably well, if we introduce a direct coupling between A and B excitons with a coupling strength of ∼50 meV. The deduced coupling strength, obtained from nonvalley-selective simulations, is in excellent agreement with the theoretical estimate for the Dexter-like intervalley A-B exciton interaction,^25^ while intravalley interactions are expected to be weaker.^27^ The simulated pump–probe map in Figure 4b matches the data in Figure 2a. The fast 11.5 fs oscillations in Δ*R*/*R* are also seen in the simulations (inset) and show similar spectral and temporal characteristics as in the experiment. By introducing EID, the simulations can qualitatively account for the spectral line shape (Figure 4c), although its effect is slightly overestimated. Further improvement in agreement may be achieved by also considering additional many-body effects, such as EIS and state filling, in the simulations. Also the coherent dynamics of the A and B exciton resonances (Figure 4d) match well with experiment, showing pronounced oscillations predominantly on the B exciton resonance. Oscillations in amplitude on the A exciton are weaker, since the overlap of the pump with the B exciton is low. Thus, the coupling-induced change in B exciton population is more pronounced than for A. The delayed rise and slower population dynamics cannot be captured, since relaxation between the exciton states is not included. The simulated 2DES map (Figure 4e) reproduces the key experimental features, i.e. the EID line shape of (A,A) and the cross peaks between A and B excitons. Vertical stripes in 2DES are suppressed, since higher-lying excitations are not included. As a result, small deviations in the relative A and B exciton peak amplitudes in Figure 4c arise. The line shape of the (A,A) diagonal peak is well reproduced when including a finite amount of inhomogeneous broadening (see SI Section 4). We deduce dephasing times of *T*_2, *A*_ = 50 fs and *T*_2, *B*_ = 20 fs for the A and B excitons, respectively, and a Gaussian inhomogeneous broadening with a standard deviation σ = 14 meV. Also the PPFID decay dynamics in pump–probe (Figure 4d) can only be reproduced when including inhomogeneous broadening.

For a microscopic analysis of the coupling between excitons residing in different valleys, numerical solutions of the SBE are used (see SI section 6). We consider the optical transitions and the electron–hole Coulomb interactions, which lead to A and B, as well as A’ and B’ excitons in the respective valleys. Additionally, we include the Coulomb interaction between A and B’, as well as A’ and B excitons (Dexter coupling).^27^ While the pump pulse predominantly generates A excitons, the Dexter coupling also drives B excitons, thereby enabling A-B’ and A’-B-exciton polarization interferences, which show up as oscillations in the time-resolved Δ*R*/*R* signal (Figure 5). The oscillation period of 11.3 fs mainly matches the A-B exciton splitting. In these simulations, Coulomb interactions can lead to a coupling between excitons with the same spin.^25,27^ Evidence for such intervalley couplings was previously presented for 1L-MoS_2_ in ref (30). Intravalley interactions between excitons with different spin^5,29^ are not included in the SBE simulations. A coupling strength of 28 meV was deduced from 2DES of 1L-MoS_2_ in ref (29) and theoretical work indicates that this coupling is weaker than the Dexter-like interaction.^27^ This suggests that the ultrafast oscillations mainly reflect Dexter-like intervalley coupling. Without helicity resolution, our experiments cannot directly distinguish between the two mechanisms, calling for new experiments that combine valley-selective excitation and broadband spectroscopy with ultrahigh time resolution.

**Figure 5:**
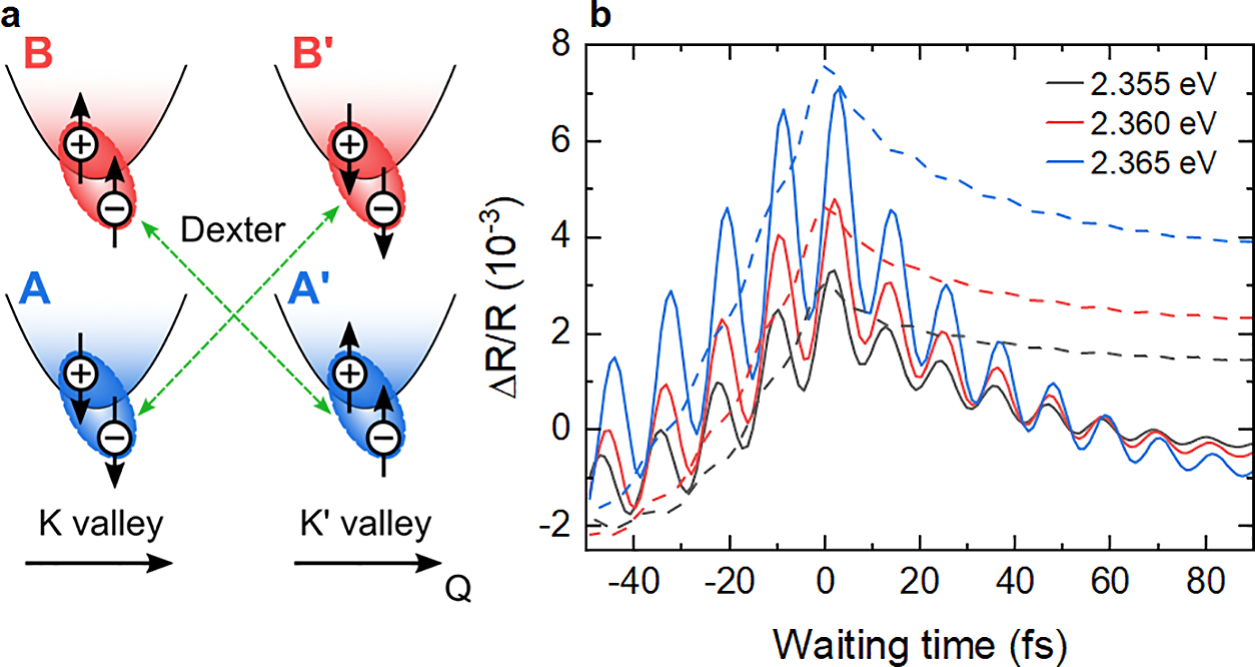
(a) Scheme of Dexter-like intervalley coupling between the A and B excitons in different valleys. (b) Calculated ΔR/R dynamics with (solid lines) and without (dashed lines) Dexter coupling. If the Dexter coupling is omitted, the A–B exciton polarization interference is strongly suppressed, as B excitons are no longer driven by the Coulomb interaction. A minimal oscillation remains due to weak residual direct optical pumping of the B excitons with the tail of the spectrally broad 5 fs pump pulse.

In summary we reported pump–probe and 2DES experiments performed with sub-10 fs resolution on 1L-WS_2_ at RT. Our results show that the optical nonlinearities are largely dominated by ultrafast many-body interactions, specifically excitation induced dephasing and shifts of the exciton resonances, in quantitative agreement with earlier studies.^11,25,27^ Our experiments provide evidence for a coherent coupling between A and B excitons in 1L-WS_2_, manifesting itself not only in the appearance of distinct cross peaks in 2DES, but also in rapid oscillations of the nonlinear signals with 11.5 fs period during the coherence time of the excitons. Comparison to microscopic SBE simulations suggest that these oscillations mainly arise from a coherent population transfer between excitons with the same spin in opposite valleys, induced by Dexter-like intervalley interactions. Intravalley couplings may also contribute, but are expected to be weaker. From our data, we estimate a coupling strength of 50 meV. Ultrafast experiments with helicity-resolved excitation may probe such intervalley population switching more directly.^65^ Pronounced vertical stripes in our 2DES spectra point to additional dephasing effects induced by the excitation of higher lying resonances. Our results suggest that ultrafast and broadband 2DES^52^ is an highly sensitive tool for studying these EID effects in layered semiconductor materials and, more generally, for probing and manipulating off-resonant coherent couplings and many-body interactions.

## Data Availability

The experimental data supporting the claims in this study are presented in the manuscript and Supporting Information in graphic form and can be obtained from the authors upon reasonable request.

## Supporting Information Available

Experimental methods, sample preparation, fluence study, line shape analysis, simulation and theory details, Figures S1–S6, and Tables S1–S2 (PDF)
