# Supplementary material for: Ultrafast Coherent Exciton Couplings and Many-Body Interactions in Monolayer WS2
Source: Nano Lett. 2024 Jun 20;24(26):8117–25. doi: 10.1021/acs.nanolett.4c01991 (PMC11229071; doi:10.1021/acs.nanolett.4c01991)
Supplement: Supplementary file 1 — nl4c01991_si_001.pdf [file nl4c01991_si_001.pdf]

Supporting Information for

Ultrafast coherent exciton couplings and many-body interactions in monolayer WS_2

Daniel Timmer[a], Moritz Gittinger[a], Thomas Quenzel[a], Alisson R. Cadore[b,c], Barbara L.T. Rosa[b], Wenshan Li[b], Giancarlo Soavi[b,d], Daniel C. Lünemann[a], Sven Stephan[a,e], Martin Silies[a,e], Tommy Schulz[f], Alexander Steinhoff[f], Frank Jahnke[f], Giulio Cerullo[g,h], Andrea C. Ferrari[b], Antonietta De Sio[a,i], and Christoph Lienau[a,i,*]

[a] Institut für Physik, Carl von Ossietzky Universität, 26129 Oldenburg, Germany

[b] Cambridge Graphene Centre, University of Cambridge, CB3 0FA Cambridge, United Kingdom

[c] Present address: Brazilian Nanotechnology National Laboratory, Brazilian Center for Research in Energy and Materials, São Paulo, Brazil.

[d] Present address: Institute of Solid State Physics, Friedrich Schiller University Jena, Max-Wien Platz 1, 07743 Jena, Germany and Abbe Center of Photonics, Friedrich Schiller University Jena, Albert-Einstein-Straße 6, 07745 Jena, Germany.

[e] Present address: Institute for Lasers and Optics, University of Applied Sciences, 26723 Emden, Germany

[f] Institute for Theoretical Physics and Bremen Center for Computational Materials Science, University of Bremen, P.O. Box 330 440, 28334 Bremen, Germany

[g] Dipartimento di Fisica, Politecnico di Milano, Piazza L. da Vinci 32, 20133 Milano, Italy

[h] Istituto di Fotonica e Nanotecnologie-CNR, Piazza L. da Vinci 32, 20133 Milano, Italy

[i] Center for Nanoscale Dynamics (CENAD), Carl von Ossietzky Universität Oldenburg, Institut für Physik, 26129 Oldenburg, Germany

*Correspondence to: christoph.lienau@uni-oldenbug.de

Table of content

1. **Experimental setup** 1
2. **Sample preparation** 4
3. **Fluence study** 4
4. **Homogeneous and inhomogeneous broadening** 5
5. **Simulation of pump-probe and 2DES data** 6
6. **Simulations based on semiconductor Bloch equations** 10
7. **References** 11

1. Experimental setup

Pump-probe and two-dimensional electronic spectroscopy (2DES) experiments are performed using a home-built, non-collinear optical parametric amplifier (NOPA). This is pumped by a fiber amplifier (Tangerine V2, Amplitude Systèmes) operating at 175 kHz and generating 260-fs pulses (Full-Width at Half-Maximum, FWHM) at 1030 nm. Fig. S1 displays the NOPA layout, which is adapted from Ref. 1. A small

fraction of the 1030-nm pump beam is directly used to generate a supercontinuum in a 4-mm-thick YAG crystal (yellow beam in Fig. S1). The remaining portion of the pump beam is converted to its third harmonic (TH) via a set of two BBO crystals. The first crystal frequency-doubles the pump beam (cut angle θ = 23.4°, thickness 3 mm) to generate the second harmonic (SH, green beam in Fig. S1). Then the SH is combined in a second BBO crystal with the remaining fundamental light (θ = 32.5°, thickness 2 mm) to generate the TH (blue beam in Fig. S1). The YAG seed and TH pump are focused into another BBO crystal (θ = 37.0°, thickness 1 mm) under an angle of 6.5° to drive a non-collinear optical parametric amplification process. In order to allow for broadband amplification, the chirped white light supercontinuum pulse is compressed to match the duration of the pump pulse using a pair of chirped mirrors (DCM9, Laser Quantum). The amplified seed beam (red beam in Fig. S1) is pre-compressed to compensate dispersion using another pair of chirped mirrors (DCM9, Laser Quantum).

The 2DES setup used for the experiments was discussed in Ref. 2. The NOPA output is split into a pump and a probe beam via a beam splitter. Afterwards, the pump beam goes through a mechanical chopper system (MC2000B, Thorlabs) with a custom-made wheel (500 slots) synchronized with the laser source. Chopping is performed at a modulation frequency of 43.75 kHz, one quarter of the repetition rate of the laser.

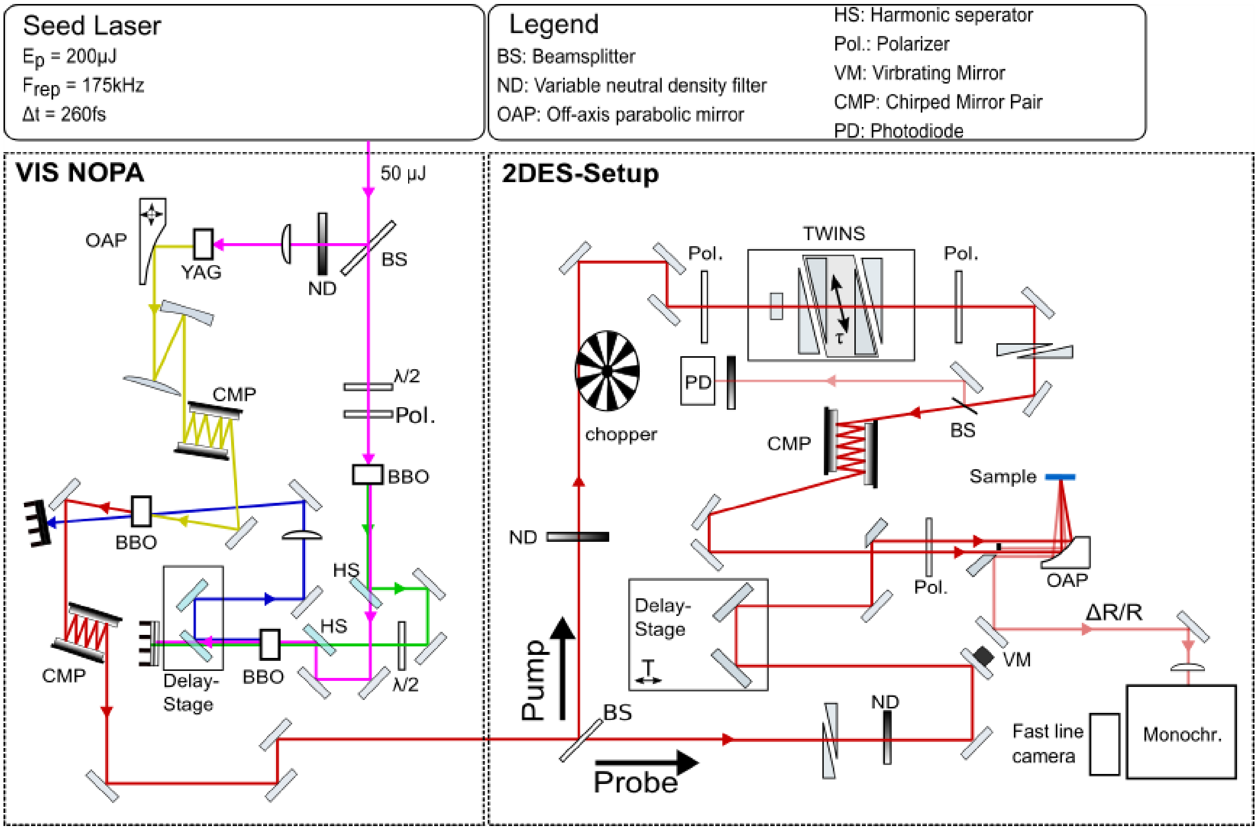

Figure S1: *Experimental 2DES setup and pulse generation. A home-built NOPA (left) is pumped with 260-fs pulses of 50 µJ energy at a center wavelength of 1030 nm and 175 kHz repetition rate. The output of the VIS-NOPA covers a spectral range from ~515-690 nm (Fig. 1c). The NOPA pulses are used in a home-built 2DES setup (right).*[2] *Key components of the 2DES setup are: (i) a passively phase-stabilized TWINS interferometer, (ii) a fast line camera detector operating at 87.5 kHz and (iii) a home-built high-repetition-rate mechanical chopper.*

Using an in-line interferometer based on birefringent wedges (TWINS, Translating-Wedge-Based Identical Pulses eNcoding System)[3] we then generate a pair of phase-stable pump pulses with tunable pulse separation (coherence time τ) to record 2DES data. A small fraction of the pump light is sent to a photo diode behind the interferometer that records a field autocorrelation for each coherence time scan. The relative delay between the pump-pulse pair and the probe can be set via a retro reflector on a motorized translation stage (M126.DG1, Physik Instrumente). This delay between the second pump pulse and the probe is denoted as the waiting time T. The pump and probe beams are focused onto the sample under a small few-degree angle using an off-axis parabolic mirror (OAP, reflected focal length of 50 mm). Both are vertically polarized. Their spot sizes are characterized with a CMOS camera to be 12x21 µm² for the probe and 19x22 µm² for the pump. The reflected beams are collimated using the same OAP. The probe beam is spectrally dispersed in a grating monochromator (Acton SP2150i, Princeton Instruments) and detected with an attached fast (up to 126 kHz) and highly sensitive line camera (Aviiva EM4, e2v).
Using the chopper wheel to periodically modulate the pump pulses in pairs of two (denoted as *on* or *off*) and the fast line camera (operating at 87.5 kHz), we record differential reflectivity spectra:

$$\frac{\Delta R}{R}(\tau, T, E_{det}) = \frac{S_{on}(\tau, T, E_{det}) - S_{off}(E_{det})}{S_{off}(E_{det})} \tag{S1}$$

with a rate of 43.75 kHz as a function of the coherence time τ, waiting time T and detection energy E_{det}, measured using the grating monochromator. In a pump-probe measurement, $\tau = 0$ fs and differential spectra are recorded as a function of T. For a 2DES measurement, a τ scan is performed for each T, and differential spectra are recorded on the fly. By performing a Fourier transform of the recorded interferogram along τ and taking the real part of this signal we obtain absorptive 2DES maps:

$$A_{2D}(E_{ex}, T, E_{det}) = \Re\left(\int_0^\infty \frac{\Delta R}{R}(\tau, T, E_{det}) e^{\frac{iE_{ex}\tau}{\hbar}} d\tau\right) \tag{S2}$$

These are energy-energy maps of the differential reflectivity as a function of the excitation energy E_{ex}, and detection energy E_{det}, recorded for each T.
A cross correlation second harmonic frequency-resolved optical gating (SH-FROG) measurement between pump ($\tau = 0$ fs) and probe at the sample position, taken with a 10-µm BBO crystal, is in Fig. S2, giving a ~9fs experimental pulse duration at the sample position. All experiments are performed at room temperature (RT).

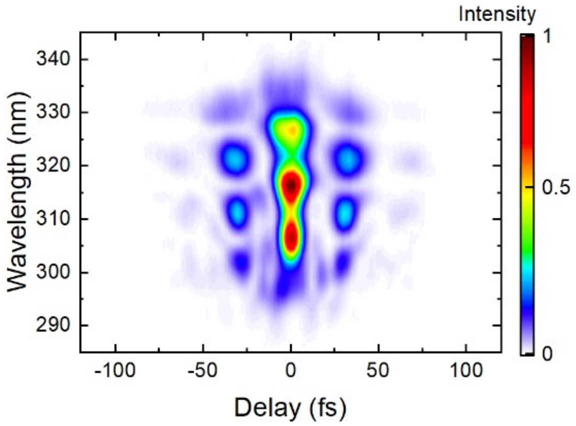

Figure S2: *SH-FROG measurement of the cross correlation between pump and probe pulses at the sample position. The retrieved pulse duration is 9 fs.*

2. Sample preparation

Polycrystalline Ag films with a thickness of 200 nm are deposited on a fused silica substrate using e-beam evaporation, then coated with a 5 nm aluminum oxide (grown at 150°C) to avoid hot electron (e) transfer between Ag and TMD.[4-6] 1L-WS_2 flakes are prepared by micro-mechanical exfoliation from bulk 2H-WS_2 (HQ Graphene source) on Nitto Denko tape,[7] and then exfoliated again on a polydimethylsiloxane (PDMS) stamp placed on a glass slide for inspection under an optical microscope. Optical contrast is optimized to identify 1L-WS_2 prior to dry transfer.[8] Selected flakes are aligned and stamped on the desired location with a micro-manipulator at 60°C, before increasing the temperature to 80°C, so the flakes detach from the PDMS and adhere preferentially to the substrate. Raman and photoluminescence (PL) spectroscopy (Fig S3) confirm that the investigated flake with a size of ~50x70 µm² is a 1L-WS_2. The Raman spectrum shows the main Raman modes at ~418.8±0.2 cm^{-1} (A'_1), ~356.8±0.2 cm^{-1} (E') and ~352.1±0.2 cm^{-1} (2LAM(A)) and 8 additional Raman modes that are expected for 1L-WS_2 under 514 nm laser excitation as discussed in Ref. 9. The difference in Raman shift between the A'_1 and the E' peak of ~62 cm^{-1} is also consistent with 1L-WS_2.[9] Fig. S3b shows a PL emission at ~616.1 nm, also consistent with 1L-WS_2.[9]

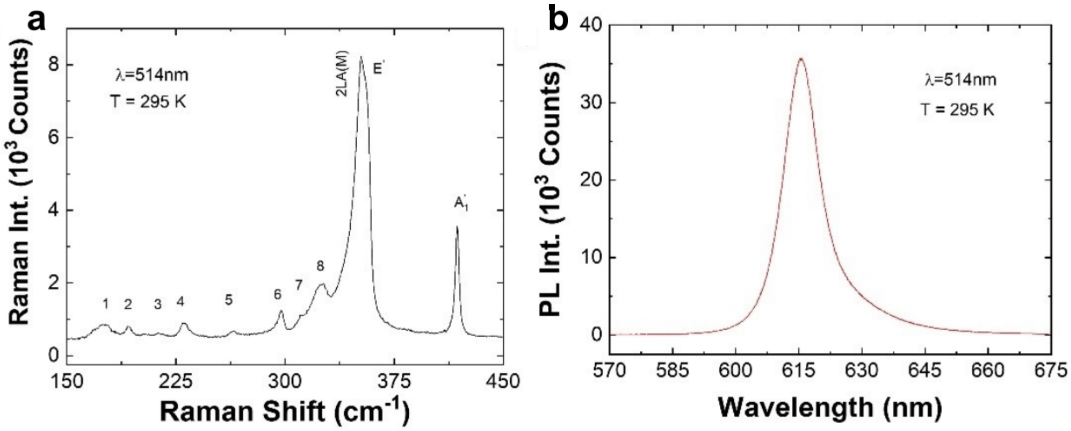

***Figure S3: a:** Raman and **b:** PL spectra of 1L-WS_2 under 514 nm excitation at RT.*

3. Fluence study

To ensure that all experiments are performed well below the Mott transition[10] and within the $\chi^{(3)}$ regime, a fluence study is conducted. Fig. S4 presents pump-probe measurements ranging from 5 to 243 µJ/cm². Within this almost two orders of magnitude variation in fluence, no significant changes to the pump-probe maps can be observed (Figs. S4a-c). A closer inspection of the lineshape and dynamics (Figs. S4d-e) reveals minor fluence-dependent changes. By increasing the incident pump power, the A exciton resonance broadens. This is consistent with our interpretation that many-body effects due to the increase in excitation density, such as EID,[11] dominate the optical nonlinearity of 1L-WS_2. In addition, the dynamics of the A exciton shows a flattening of the initial decay with increased fluence, but no changes of the early, ~100-fs dynamics. No indications of exciton-exciton annihilation processes or changes to the delayed rise of the pump-probe signal are seen. The most striking effect of the increase in fluence is shown in Fig. S4f. The integrated pump-probe signal at the A exciton resonance for $T > 150$ fs has a strong deviation from a linear behavior for fluences >30 µJ/cm², and even a saturation of the signal strength at ~200 µJ/cm². This might reflect the influence of many-body effects, such as EID,[12] rather than going beyond the $\chi^{(3)}$ regime. The experiments in the main manuscript are performed at a pump fluence of ~30 µJ/cm² and a probe fluence of ~20 µJ/cm², with the latter being sufficiently weak to not affect the observed pump-probe signal.

The ultrafast coherent oscillations observed at the B exciton resonance in Fig. 2c are also seen up to the maximum fluence of 243 µJ/cm² (Fig. S4c).

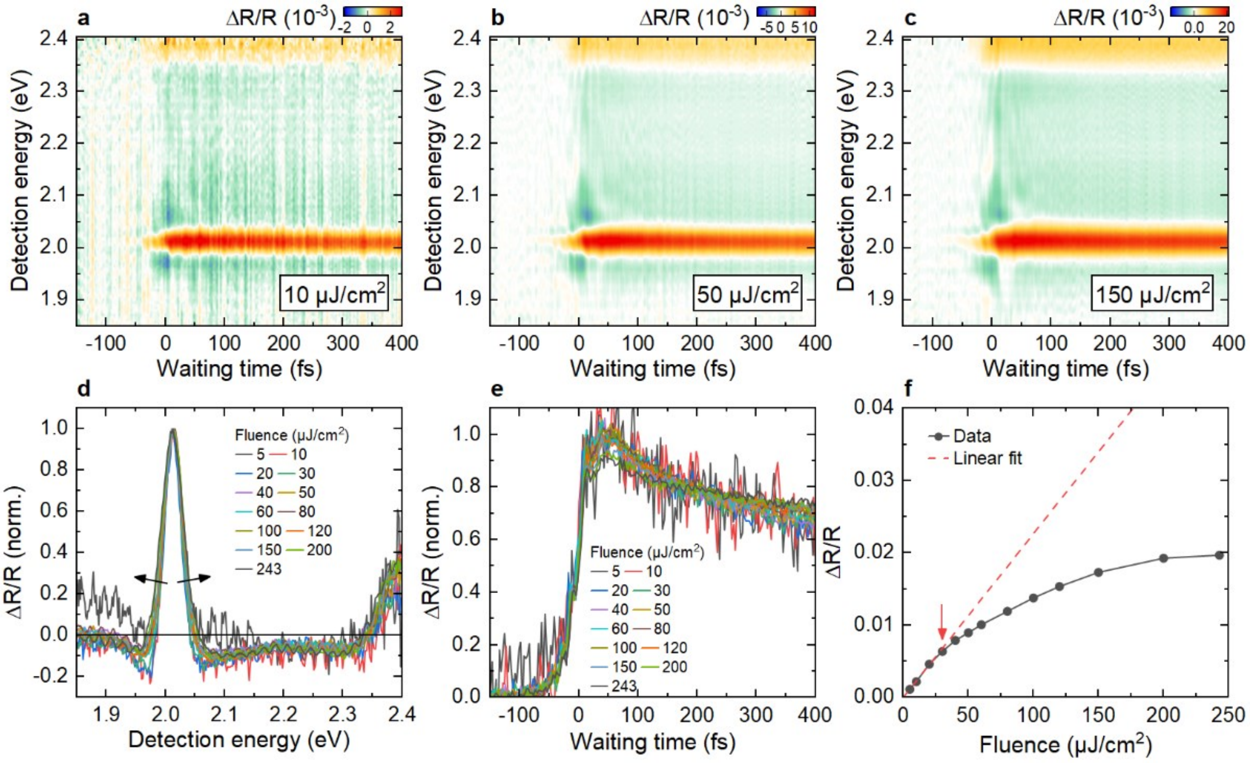

__Figure S4:__ Fluence study of the pump-probe maps recorded on 1L-WS$_2$ at RT. ***a-c:*** *Pump-probe maps at (a) 10, (b) 50, (c) 150 µJ/cm².* ***d:*** *Spectral crosscuts at T = 200 fs. A broadening of the A exciton resonance with increasing pump fluence due to EID can be seen.* ***e:*** *Dynamics at the A exciton resonance. For high fluences >100 µJ/cm² a slight slow-down of the initial fast decay component is observed, with no signatures of exciton-exciton annihilation.* ***f:*** *Average pump-probe signal for T > 150 fs. The pump-probe signal saturates at ~200 µJ/cm², with a deviation from a linear fit for >40 µJ/cm². Experiments are performed at a pump fluence of ~30 µJ/cm², corresponding to an excitation density of* $\sim 1.7 \cdot 10^{12}$ *cm*$^{-2}$*.*

To estimate the excitation density, we take into account the laser spectrum and the linear reflectivity of the sample (Fig. 1c). We can thus estimate the number of photons remaining in the sample. By taking the photon energy and pump fluence we estimate an excitation density of $\sim 1.7 \cdot 10^{12}$ cm^{-2}.

4. Homogeneous and inhomogeneous broadening

As seen from the experimental 2DES maps (Fig. 3), the sample shows signatures of an inhomogeneous broadening contribution to the exciton linewidths, especially noticeable for the diagonal A exciton peak. Using the 2DES data we can quantify the homogeneous and inhomogeneous contribution to the lineshape using the diagonal and crossdiagonal peak profile.[13] Fig. S5 presents these crosscuts for the diagonal A exciton feature for $T = 0$ fs. A Gaussian fit to the experimental profiles (black and blue lines) yields FWHM of ~16 and 33 meV for the crossdiagonal and diagonal curves, respectively. The crossdiagonal lineshape is strongly affected by EID, thus the value resulting from the fit represents a lower limit for the homogeneous broadening. While the crossdiagonal profile usually is a marker for the homogeneous linewidth and therefore the dephasing rate γ_X of the exciton, the diagonal profile marks ensemble effects due to the finite focus size such as local strain[13] or disorder[11] in 1L-TMDs. Therefore, the sample does exhibit a finite amount of inhomogeneous broadening.

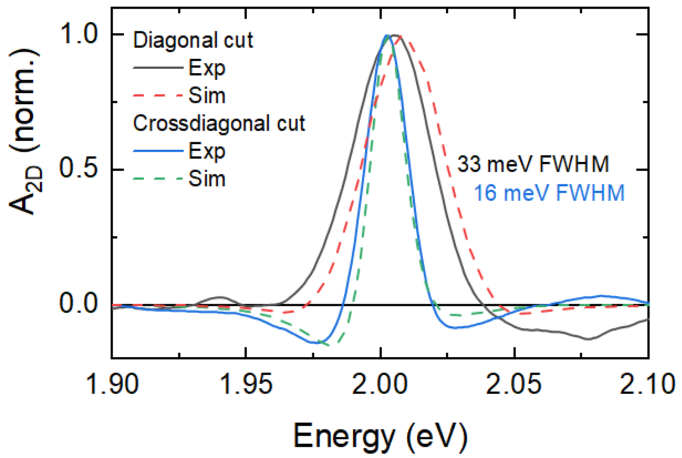

Figure S5: *Estimating homogeneous and inhomogeneous FWHM by comparing diagonal and crossdiagonal cuts through the 2DES map at T = 0 fs for experiments (black and blue solid lines) and simulations (red and green dashed lines).*

We account for the effect of experimentally observed inhomogeneity in exciton energy in the pump-probe and 2DES simulations (see section 5), using a pure dephasing time for the $X = A, B$ excitons:

$$T^*_{2,X} = 1/\gamma_X \tag{S3}$$

with dephasing rate γ_X. We use $T^*_{2,A} = 50$ fs and $T^*_{2,B} = 20$ fs to reach good agreement with the experimental data. This corresponds to $\hbar\gamma_A = 13.2$ meV and $\hbar\gamma_B = 32.9$ meV, in agreement with theoretical predictions.[14]

5. Simulation of pump-probe and 2DES data

To support the interpretation of our experimental pump-probe and 2DES data, we compute the time-dependent density matrix $\hat{\rho}$ of our system upon interaction with the electric fields of up to three ultrashort laser pulses in order to calculate the sample polarization in the time-domain.[15] For this, we numerically solve the master equation in Lindblad form:[16, 17]

$$\dot{\hat{\rho}} = -\frac{i}{\hbar}[\hat{H}, \hat{\rho}] + \frac{1}{2}\sum_k \left(2\hat{L}_k\hat{\rho}\hat{L}_k^\dagger - \hat{L}_k^\dagger\hat{L}_k\hat{\rho} - \hat{\rho}\hat{L}_k^\dagger\hat{L}_k\right). \tag{S4}$$

to obtain the evolution of the density matrix $\hat{\rho}$ under the total Hamiltonian $\hat{H} = \hat{H}_S + \hat{H}_{int}(t)$, given as the sum of the system Hamiltonian $\hat{H}_S$ and the light-matter interaction Hamiltonian $\hat{H}_{int}(t)$. System-bath interactions, such as population relaxation and dephasing phenomena, are accounted for via Lindblad operators $\hat{L}_k$.[16, 17]

In this non-perturbative approach,[18-20] the system interaction with the coherent laser pulses can be described in the dipolar approximation,[21, 22] via the interaction Hamiltonian $\hat{H}_{int}(t) = -\hat{\mu}E(t)$ that couples the transition dipole moment operator $\hat{\mu}$ with the electric field:[23]

$$E(t) = \sum_{\substack{n=pu1,\\ pu2,pr}} E_{0,n} e^{-2ln2\left(\frac{t-t'_n}{\Delta t}\right)^2} \cos(\omega_L(t - t'_n) + \phi_n). \tag{S5}$$

This total electric field consists of up to three Gaussian laser pulses (pump 1, pump 2 and probe) with amplitude $E_{0,n}$, pulse duration $\Delta t = 5$ fs and frequency $\omega_L = 2.15$ eV/$\hbar$. Additionally, the phases ϕ_n can be set for phase cycling, and we can shift the pulses in time via t'_n. We label the delay between the two pump pulses as $= t'_{pu2} - t'_{pu1}$, and the delay between the second pump pulse and the probe pulse as $T = t'_{pr} - t'_{pu2}$. The time t is the detection time, defined to be zero at the arrival time of the probe pulse, and

is used for the numerical integration of Eq. (S4). From this numerical integration we obtain the time-dependent density matrix $\hat{\rho}(t)$, which allows us to calculate the time-domain polarization:[21, 22]

$$P(t) = Tr\big(\hat{\mu}\hat{\rho}(t)\big). \tag{S6}$$

as the trace of the expectation value of the transition dipole moment operator. To isolate the signals corresponding to the linear and nonlinear data measured in the experiment, we calculate both linear $P^{(1)}(t)$ (pump *off*) and total polarization $P^{tot}(\tau, T, t)$ (pump *on*). From these, the linear and total susceptibilities can be derived as:[24]

$$\chi^{(1)}(E_{det}) = \frac{1}{\varepsilon_0}\mathcal{F}\big(P^{(1)}(t)\big)/\mathcal{F}\big(E_{pr}(t)\big) \tag{S7}$$

$$\chi^{tot}(\tau, T, E_{det}) = \frac{1}{\varepsilon_0}\mathcal{F}(P^{tot}(\tau, T, t))/\mathcal{F}\big(E_{pr}(t)\big) \tag{S8}$$

via Fourier transforms along the detection time using the vacuum dielectric constant ε_0. We thus obtain E_{det}. The total susceptibility includes all possible interactions with the three laser pulses, thus also signals not measured in the experiment, since they are not radiated into the probe direction. To account for the experimental selectivity via directional phase matching, we employ a 4 step phase-cycling scheme in the simulations.[18-20] Here, $P^{tot}(\tau, T, t)$ is repeatedly calculated for 4 phase settings $\phi_{pu1} = \phi_{pu2} = \left[0, \frac{\pi}{2}, \pi, \frac{3\pi}{2}\right]$, keeping $\phi_{pr} = 0$. By averaging these, we obtain $\chi_{PC}^{tot}(\tau, T, E_{det})$ and can isolate the experimentally accessible nonlinear susceptibility[25]

$$\chi^{nl}(\tau, T, E_{det}) = \chi_{PC}^{tot}(\tau, T, E_{det}) - \chi^{(1)}(E_{det}) \tag{S9}$$

by removing the linear contribution from the total phase-cycled susceptibility. Although the experiments are performed in a reflection geometry, we probe absorptive lineshapes for the excitons since the 1L-WS_2 is placed on a Ag mirror. The linear reflectivity therefore follows the imaginary part of the sample susceptibility. Thus, we take the imaginary part of the complex nonlinear susceptibility as our 2DES signal:[16, 17]

$$S_{2D}(\tau, T, E_{det}) = \Im\big(\chi^{nl}(\tau, T, E_{det})\big). \tag{S10}$$

By Fourier transforming this interferogram along τ and taking the real part we obtain the absorptive energy-energy 2DES maps: [16, 17]

$$S_{2D}(E_{ex}, T, E_{det}) = \Re\left(\int_{-\infty}^{\infty} \theta(\tau) S_{2D}(\tau, T, E_{det}) e^{iE_{ex}\tau/\hbar} d\tau\right) \tag{S11}$$

as a function of $E_{det}$and $E_{ex}$for each T. Causality is enforced via the Heaviside step function $\theta(\tau)$.

System Hamiltonian and Lindblad Terms

We set up the system Hamiltonian:

$$\hat{H}_S = E_A\hat{b}_A^\dagger\hat{b}_A + E_B\hat{b}_B^\dagger\hat{b}_B + \hat{H}_{AB} \tag{S12}$$

by employing exciton creation and annihilation operators $\hat{b}_X^\dagger$ and $\hat{b}_X$, respectively. Interactions between A and B excitons are accounted for by the coupling Hamiltonian in the rotating wave approximation:[25]

$$\hat{H}_{AB} = V_{AB}\big(\hat{b}_A^\dagger\hat{b}_B + \hat{b}_B^\dagger\hat{b}_A\big) \tag{S13}$$

with coupling strength V_{AB}. The exciton transition dipole moment operator reads:

$$\hat{\mu}_X = \mu_X\big(\hat{b}_X^\dagger + \hat{b}_X\big). \tag{S14}$$

The population relaxation of the excitons is introduced using Lindblad operators:[25]

$$\hat{L}_{rel,X} = \sqrt{\kappa_X}\hat{b}_X \tag{S15}$$

with exciton lifetime $T_{1,X} = 1/\kappa_X$. Similarly, we introduce dephasing via:[25]

$$\hat{L}_{dep,X} = \sqrt{2\gamma_X}\hat{b}_X^\dagger \hat{b}_X. \tag{S16}$$

Here, each class of excitons (A, B) interacts with independent baths via an individual Lindblad operator.

Modeling of many-body effects

The many-body Hamiltonian introduced here (see Fig. 4a) contains two 1-quantum (1Q) states $|X_A\rangle$ and $|X_B\rangle$ and three two-quantum (2Q) states $|X_AX_A\rangle$, $|X_BX_B\rangle$ and $|X_AX_B\rangle$. The mixed $|X_AX_B\rangle$ state suppresses 2DES cross peaks and coherent oscillations via the collective ground state. This is achieved by canceling ground state bleaching (GSB) and stimulated emission (SE) pathways with ESA of opposite signs. The 2Q excitations $|X_AX_A\rangle$ and $|X_BX_B\rangle$ are introduced to phenomenologically account for many-body effects, [26] such as EIS, EID, or a finite bleaching of the transitions from the 1Q to the 2Q manifold. These 2Q states do not reflect bound biexciton states. In this framework, EIS is reflected by a finite energy difference between the 2Q state and the sum of the constituent 1Q excitations. EID implies an increase in dephasing when going from 1Q to 2Q states, while Pauli blocking can be accounted for by reducing the amplitude of the 1Q→2Q transition dipole moment. All three mechanisms affect the balance between 1Q and 2Q transitions and transform the Hamiltonian from a linear to nonlinear one in the applied optical field.

From the experiments, we estimate that EID dominates the nonlinearity of the sample, therefore we introduce this as the microscopic source of the nonlinearity in our simulations. Qualitatively similar results are also obtained when adding a finite amount of EIS or bleaching. The density-dependent linewidth broadening[11] and saturation of the nonlinearity seen in the fluence-dependent study of Fig. S4 are not covered by our Hamiltonian. They would require density-dependent many-body interactions and/or the inclusion of higher-lying quantum states.

To account for EID effects, we modify the system-bath interactions via $\hat{L}_{dep,X}$. After this modification, the altered terms in the operators read:

$$\begin{aligned} \langle X_AX_A|\hat{L}_{dep,A}|X_AX_A\rangle &= 2\sqrt{2\gamma_A'} \\ \langle X_BX_B|\hat{L}_{dep,B}|X_BX_B\rangle &= 2\sqrt{2\gamma_B'} \\ \langle X_AX_B|\hat{L}_{dep,A}|X_AX_B\rangle &= \sqrt{2\gamma_A''} \\ \langle X_AX_B|\hat{L}_{dep,B}|X_AX_B\rangle &= \sqrt{2\gamma_A''} \end{aligned} \tag{S17}$$

where γ_X have been replaced by modified rates γ_X' in the first two and γ_X'' in the last two cases.

These four terms represent two different EID mechanisms. The first two lines of Eq. (S17) alter the system-bath interactions of the $|X_AX_A\rangle$ and $|X_BX_B\rangle$ state, thus reflect intra-species (A-A or B-B) manybody interactions. These terms are clearly required to reproduce the lineshape of the diagonal features of the excitons in the 2DES maps in Fig. 3. This EID mechanism neither introduces (A,B) or (B,A) cross-peaks in a 2DES map nor coherent oscillations in the dynamics.

The second case, the bottom two lines in Eq. (S17), describes inter-species interactions, where an A exciton population increases the dephasing of the B exciton transition and vice versa. This interaction directly affects the mixed $|X_AX_B\rangle$ 2Q state responsible for suppressing signatures of A-B exciton coherences via ESA transitions. Thus, changing the amplitude, dephasing or energy of the transition from 1Q states to $|X_AX_B\rangle$ has a similar effect as introducing a direct coupling between A and B excitons. Inter-species EID can introduce cross-peaks and coherent exciton oscillations in the nonlinear experiment without the presence of A-B exciton populations, due to a lack of explicit A-B coupling. Such a model was used in Ref. 27 to describe a possible effect of EID and EIS on cross-peaks between A and B excitons in 2DES maps of 1L-MoS_2. In Ref. 27, a comparison between experiment and simulation suggested that this effect could not

explain the observed cross-peaks and their dynamics. Instead, a direct coupling between A and B excitons of ~28 meV was found to reproduce the experimental observations.

To simulate our experiments, we introduce only the first class of intra-species EID. Coherent couplings between A and B excitons are taken into account by a direct coupling term V_{AB}, rather than by introducing indirect coupling via an inter-species EID or EIS. Such a phenomenological model reproduces our key experimental observations. It contains several free parameters to account for many-body nonlinearities. While the chosen parameters seem realistic, based on existing microscopic calculations,[28] this does not imply that other sets of parameters may not give similar or better agreement with experiments.

Simulation parameters

We set up the system assuming the same transition dipole moments for both A and B excitons, $\mu_A = \mu_B$, with $\gamma_X'' = \gamma_X$, so that no effect of inter-species EID is introduced. We further add a fixed amount of intra-species EID by setting $\gamma_X' = 1.1\gamma_X$, increasing the homogeneous linewidth by 10% (Fig. S6). All additional simulation parameters are listed in Table S1.

***Table S1**: Parameters used in the simulations.*

Parameter	Value
E_A	2.014 eV
E_B	2.384 eV
V_{AB}	-50 meV
$T_{1,X}$	8 ps
$T_{2,A}^*$	50 fs
$T_{2,B}^*$	20 fs

The Hamiltonian (Fig. 3a) only contains states up to the 2Q manifold. The light-matter interaction energy μE_0 is kept sufficiently weak to only introduce a depletion of the ground state population on the order of 10^{-7} after excitation with both pump pulses ($\tau = 0$).

Inhomogeneous broadening effects

To account for inhomogeneous broadening, the time-dependent simulations of the master equation are repeated while varying the energies of the A and B excitons by an ΔE ranging from -30 to 30 meV in steps of 10 meV. A weighted averaging of these 7 pump-probe and 2DES maps then introduces inhomogeneous broadening, giving the simulations of Fig. 4. For this, we determine a Gaussian distribution with $\sigma = 14$ meV standard deviation. Crosscuts of the simulated 2DES map at $T = 0$ fs are in Fig. S5 (dashed lines), in good agreement with experiments (solid lines). A small energy shift of ~5 meV between the diagonal profiles of experiments and simulations reflects a finite displacement of the A exciton feature from the diagonal position, not captured by the simulation. Experiments and simulations show good agreement for the crossdiagonal profiles. The deduced value for $\hbar\gamma_A \approx 13.2$ meV gives a homogeneous FWHM of ~26.4 meV in linear reflectivity. This is larger than the ~16 meV linewidth seen in the crossdiagonal 2DES lineshape (Fig. S5). This apparent narrowing is a consequence of EID as the dominant source of exciton nonlinearity. The pronounced narrowing effect of EID on the differential reflectivity profile is exemplarily shown in Fig. S6 for a Lorentzian without inhomogeneous broadening contribution.

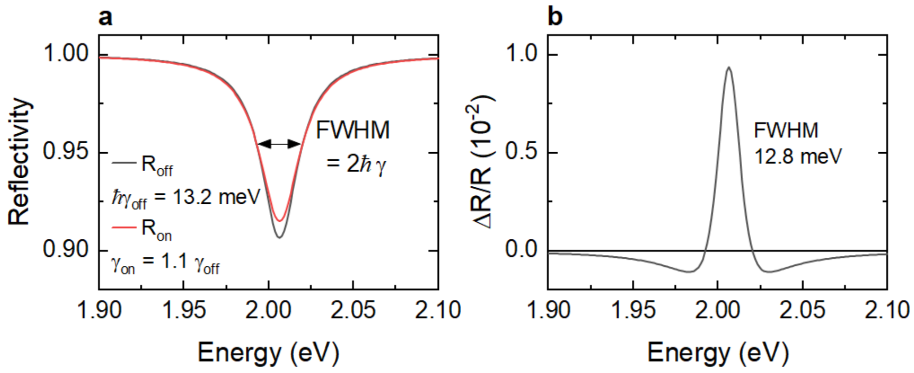

***Figure S6**: Effect of excitation-induced dephasing on reflectivity.* ***a**: Simulated reflectivity for the A exciton assuming a homogeneous linewidth that corresponds to a dephasing time of ~50 fs (black line) and a line-broadening by ~10% via EID (red line).* ***b**: Differential reflectivity as calculated from panel a. The FWHM of ~12.8 meV is much smaller than that of the absorptive linear reflectivity of ~26.4 meV.*

6. Simulations based on semiconductor Bloch equations

To analyze the role of Dexter coupling microscopically, we use the semiconductor Bloch equations[29] (SBE) for the carrier occupation probabilities $f_{\mathbf{k}\xi s}(t)$ and the interband transition amplitudes $\psi_{\mathbf{k}\xi s}(t)$:

$$i\hbar \frac{\mathrm{d}\psi_{\mathbf{k}\xi s}(t)}{\mathrm{d}t} = \left[E^{g}_{\xi s} + \frac{\hbar^2 k^2}{2\mu_{\xi s}} + \Sigma_{\mathbf{k}\xi s}(t) - i\Gamma\right]\psi_{\mathbf{k}\xi s}(t) - \left[1 - 2f_{\mathbf{k}\xi s}(t)\right]\Omega_{\mathbf{k}\xi s}(t),$$
$$i\hbar \frac{\mathrm{d}f_{\mathbf{k}\xi s}(t)}{\mathrm{d}t} = \Omega^{*}_{\mathbf{k}\xi s}(t)\psi_{\mathbf{k}\xi s}(t) - \Omega_{\mathbf{k}\xi s}(t)\psi^{*}_{\mathbf{k}\xi s}(t). \qquad \text{(S18)}$$

Here, we consider optically driven interband transitions between the topmost valence band and the lowest conduction band of a 1L-TMD semiconductor. The states of electrons and holes are described by the carrier wave vector $\mathbf{k}$ centered around the high-symmetry point $\xi = (K, K')$ and spin $s = (\uparrow, \downarrow)$. $E^{g}_{\xi s}$ is the bandgap energy and with the effective masses of electrons and holes, $m^{e/h}_{\xi s}$, the reduced mass is introduced via $1/\mu_{\xi s} = 1/m^{\mathrm{e}}_{\xi s} + 1/m^{\mathrm{h}}_{\xi s}$. The dephasing of exciton states is described via Γ. Furthermore,

$$\Sigma_{\mathbf{k}\xi s}(t) = -2\sum_{\mathbf{k}',\xi'} V_{\left|\Delta_{\xi\xi'}+\mathbf{k}-\mathbf{k}'\right|}\, f_{\mathbf{k}'\xi' s}(t) \qquad \text{(S19)}$$

accounts for renormalizations of the electron and hole energies. The factor two in (S18) and (S19) appears due to the degeneration of the electron and hole occupation probabilities. Moreover,

$$\Omega_{\mathbf{k}\xi s}(t) = d_{\mathbf{k}\xi s}E(t) + \sum_{\mathbf{k}',\xi} V_{\left|\Delta_{\xi\xi'}+\mathbf{k}-\mathbf{k}'\right|}\, \psi_{\mathbf{k}'\xi' s}(t) \qquad \text{(S20)}$$

is the generalized Rabi energy. It contains the optical driving term of interband transitions via the dipole coupling $d_{\mathbf{k}\xi s}$ and the optical field $E(t)$. For the former we employ an analytical expression from a simple two-band model derived in Ref. 30. The Coulomb matrix element is given by $V_{|\mathbf{q}|} = U_{|\mathbf{q}|}/\varepsilon_{|\mathbf{q}|}$ where $U_{|\mathbf{q}|}$ is the bare Coulomb interaction and $\varepsilon_{|\mathbf{q}|}$ the dielectric screening due to the environment and the monolayer itself. For the latter an analytical expression derived by Ref. 31 is used, which describes a TMD layer of a finite width separated from a substrate below by an airgap h_{int}. In (S19) and (S20) the terms with $\xi' = \xi$ describe intravalley Coulomb interaction while those with $\xi' \neq \xi$ account for intervalley interaction which

is referred to as Dexter coupling. Furthermore, $\mathbf{\Delta}_{\xi\xi'}$ is the vector connecting the valleys ξ and ξ' and $|\Delta_{KK'}| = \frac{4\,\pi}{3\,a_0}$ with the lattice constant a_0.

A numerical solution of (S18)-(S20) provides the macroscopic polarization

$$P(t) = \frac{1}{\mathcal{A}} \sum_{\mathbf{k}\xi s} d^{*}_{\mathbf{k}\xi s} \psi_{\mathbf{k}\xi s}(t) \tag{S21}$$

and the optical susceptibility

$$\chi(\omega) = \frac{1}{\varepsilon_0} \frac{P_0(\omega)}{E(\omega)}. \tag{S22}$$

Here $P_0(\omega)$ is the component of $P(\omega)$ in probe direction that is obtained by phase cycling[19, 32] analogous to the procedure also used in section 5 of the supplement. The sample reflectivity $R(\omega)$ follows from the susceptibility and a solution of Maxwell's equations. The light propagation through the TMD and sapphire as well as the reflection at the silver surface are incorporated via the transfer matrix formalism.[33] The parameters used in the simulation can be found in Table S2.

***Table S2**: Material constants used for the numerical solution of the SBE. The effective electron and hole masses are in units of the free electron mass m_0. The values of the gap energies and dephasing are chosen to match theory and experiment.*

Description	Symbol	Value	Source
Electron mass	$m^{\mathrm{e}}_{K\uparrow}, m^{\mathrm{e}}_{K\downarrow}$ (m_0)	0.27, 0.36	Ref. 34
Hole mass	$m^{\mathrm{h}}_{K\uparrow}, m^{\mathrm{h}}_{K\downarrow}$ (m_0)	0.36, 0.50	Ref. 34
Gap energy	$E^{g}_{\xi s}$ (meV)	2506, 2947	
Dephasing	Γ (meV)	10	
Lattice constant	a_0 (nm)	0.312	Ref. 35
TMD dielectric constant	$\varepsilon_{\mathrm{TMD},\perp}$, $\varepsilon_{\mathrm{TMD},\parallel}$	13.6, 6.3	Ref. 35
Sapphire dielectric constant	$\varepsilon_{\mathrm{sapphire}}$	8.6	Ref. 36
Sapphire refractive index	n_{sapphire}	1.76	Ref. 36
TMD thickness	h_{TMD} (nm)	0.614	Ref. 35
Airgap thickness	h_{int} (nm)	0.5	Ref. 31
Sapphire thickness	h_{sapphire} (nm)	5	

7. References

(1) Grupp, A.; Budweg, A.; Fischer, M. P.; Allerbeck, J.; Soavi, G.; Leitenstorfer, A.; Brida, D. Broadly tunable ultrafast pump-probe system operating at multi-kHz repetition rate. *Journal of Optics* **2018**, *20* (1), 014005.
(2) Quenzel, T.; Timmer, D.; Gittinger, M.; Zablocki, J.; Zheng, F. L.; Schiek, M.; Lützen, A.; Frauenheim, T.; Tretiak, S.; Silies, M.; Zhong, J. H.; De Sio, A.; Lienau, C. Plasmon-Enhanced Exciton Delocalization in Squaraine-Type Molecular Aggregates. *Acs Nano* **2022**, *16* (3), 4693-4704.
(3) Brida, D.; Manzoni, C.; Cerullo, G. Phase-locked pulses for two-dimensional spectroscopy by a birefringent delay line. *Optics Letters* **2012**, *37* (15), 3027-3029.
(4) Brongersma, M. L.; Halas, N. J.; Nordlander, P. Plasmon-induced hot carrier science and technology. *Nature Nanotechnology* **2015**, *10* (1), 25-34.
(5) Li, Z.; Xiao, Y.; Gong, Y.; Wang, Z.; Kang, Y.; Zu, S.; Ajayan, P. M.; Nordlander, P.; Fang, Z. Active Light Control of the MoS2 Monolayer Exciton Binding Energy. *Acs Nano* **2015**, *9* (10), 10158-10164.

(6) Xu, C.; Yong, H. W.; He, J.; Long, R.; Cadore, A. R.; Paradisanos, I.; Ott, A. K.; Soavi, G.; Tongay, S.; Cerullo, G. Weak distance dependence of hot-electron-transfer rates at the interface between monolayer MoS2 and gold. *Acs Nano* **2020**, *15* (1), 819-828.
(7) Novoselov, K. S.; Jiang, D.; Schedin, F.; Booth, T.; Khotkevich, V.; Morozov, S.; Geim, A. K. Two-dimensional atomic crystals. *Proceedings of the National Academy of Sciences* **2005**, *102* (30), 10451-10453.
(8) Casiraghi, C.; Hartschuh, A.; Lidorikis, E.; Qian, H.; Harutyunyan, H.; Gokus, T.; Novoselov, K. S.; Ferrari, A. Rayleigh imaging of graphene and graphene layers. *Nano Letters* **2007**, *7* (9), 2711-2717.
(9) Cadore, A. R.; Rosa, B. L. T.; Paradisanos; Mignuzzi, S.; De Fazio, D.; Alexeev, E. M.; Dagkli, A.; Muench, J. E.; Kakavelakis, G.; Shinde, S. M.; Yoon, D.; Tongay, S.; Watanabe, K.; Taniguchi, T.; Lidorikis, E.; Goykhman; Soavi, G.; Ferrari, A. C. Monolayer WS2 electro- and photo-luminescence enhancement by TFSI treatment. *2d Materials* **2024**, *11* (2).
(10) Chernikov, A.; Ruppert, C.; Hill, H. M.; Rigosi, A. F.; Heinz, T. F. Population inversion and giant bandgap renormalization in atomically thin WS2 layers. *Nature Photonics* **2015**, *9* (7), 466-470.
(11) Moody, G.; Dass, C. K.; Hao, K.; Chen, C. H.; Li, L. J.; Singh, A.; Tran, K.; Clark, G.; Xu, X. D.; Berghauser, G.; Malic, E.; Knorr, A.; Li, X. Q. Intrinsic homogeneous linewidth and broadening mechanisms of excitons in monolayer transition metal dichalcogenides. *Nature Communications* **2015**, *6*, 8315.
(12) Shacklette, J. M.; Cundiff, S. T. Nonperturbative transient four-wave-mixing line shapes due to excitation-induced shift and excitation-induced dephasing. *Journal of the Optical Society of America B-Optical Physics* **2003**, *20* (4), 764-769.
(13) Purz, T. L.; Martin, E. W.; Holtzmann, W. G.; Rivera, P.; Alfrey, A.; Bates, K. M.; Deng, H.; Xu, X. D.; Cundiff, S. T. Imaging dynamic exciton interactions and coupling in transition metal dichalcogenides. *Journal of Chemical Physics* **2022**, *156* (21), 214704
(14) Bernal-Villamil, I.; Berghauser, G.; Selig, M.; Niehues, I.; Schmidt, R.; Schneider, R.; Tonndorf, P.; Erhart, P.; de Vasconcellos, S. M.; Bratschitsch, R.; Knorr, A.; Malic, E. Exciton broadening and band renormalization due to Dexter-like intervalley coupling. *2d Materials* **2018**, *5* (2), 025011.
(15) Timmer, D.; Zheng, F.; Gittinger, M.; Quenzel, T.; Lunemann, D. C.; Winte, K.; Zhang, Y.; Madjet, M. E.; Zablocki, J.; Lutzen, A.; Zhong, J. H.; De Sio, A.; Frauenheim, T.; Tretiak, S.; Lienau, C. Charge Delocalization and Vibronic Couplings in Quadrupolar Squaraine Dyes. *J Am Chem Soc* **2022**, *144* (41), 19150-19162.
(16) Palmieri, B.; Abramavicius, D.; Mukamel, S. Lindblad equations for strongly coupled populations and coherences in photosynthetic complexes. *Journal of Chemical Physics* **2009**, *130* (20).
(17) Breuer, H.-P.; Petruccione, F. *The theory of open quantum systems*; Oxford University Press, 2002.
(18) Egorova, D.; Gelin, M. F.; Domcke, W. Analysis of cross peaks in two-dimensional electronic photon-echo spectroscopy for simple models with vibrations and dissipation. *Journal of Chemical Physics* **2007**, *126* (7).
(19) Seidner, L.; Stock, G.; Domcke, W. Nonperturbative Approach to Femtosecond Spectroscopy - General-Theory and Application to Multidimensional Nonadiabatic Photoisomerization Processes. *Journal of Chemical Physics* **1995**, *103* (10), 3998-4011.
(20) Yan, S. X.; Tan, H. S. Phase cycling schemes for two-dimensional optical spectroscopy with a pump-probe beam geometry. *Chemical Physics* **2009**, *360* (1-3), 110-115.
(21) Hamm, P.; Zanni, M. T. *Concepts and methods of 2d infrared spectroscopy*; Cambridge University Pres, 2011.
(22) Mukamel, S. *Principles of nonlinear optical spectroscopy*; Oxford University Press, 1995.
(23) Träger, F. *Springer handbook of lasers and optics*; Springer, 2012.
(24) Boyd, R. *Nonlinear Optics*; Academic Press, 2008 (Third Edition).
(25) Timmer, D.; Gittinger, M.; Quenzel, T.; Stephan, S.; Zhang, Y.; Schumacher, M. F.; Lützen, A.; Silies, M.; Tretiak, S.; Zhong, J. H.; De Sio, A.; Lienau, C. Plasmon mediated coherent population oscillations in molecular aggregates. *Nature Communications* **2023**, *14* (1), 8035.
(26) Nardin, G.; Moody, G.; Singh, R.; Autry, T. M.; Li, H. B.; Morier-Genoud, F.; Cundiff, S. T. Coherent Excitonic Coupling in an Asymmetric Double InGaAs Quantum Well Arises from Many-Body Effects. *Physical Review Letters* **2014**, *112* (4), 046402.
(27) Guo, L.; Wu, M.; Cao, T.; Monahan, D. M.; Lee, Y. H.; Louie, S. G.; Fleming, G. R. Exchange-driven intravalley mixing of excitons in monolayer transition metal dichalcogenides. *Nature Physics* **2019**, *15* (3), 228-232.
(28) Katsch, F.; Selig, M.; Knorr, A. Theory of coherent pump-probe spectroscopy in monolayer transition metal dichalcogenides. *2d Materials* **2020**, *7* (1), 015021.

(29) Haug, H.; Koch, S. W. *Quantum theory of the optical and electronic properties of semiconductors*; World Scientific Publishing Company, 2009.
(30) Berkelbach, T. C.; Hybertsen, M. S.; Reichman, D. R. Bright and dark singlet excitons via linear and two-photon spectroscopy in monolayer transition-metal dichalcogenides. *Physical Review B* **2015**, *92* (8).
(31) Florian, M.; Hartmann, M.; Steinhoff, A.; Klein, J.; Holleitner, A. W.; Finley, J. J.; Wehling, T. O.; Kaniber, M.; Gies, C. The Dielectric Impact of Layer Distances on Exciton and Trion Binding Energies in van der Waals Heterostructures. *Nano Letters* **2018**, *18* (4), 2725-2732.
(32) Wang, H. B.; Thoss, M. Nonperturbative simulation of pump-probe spectra for electron transfer reactions in the condensed phase. *Chemical Physics Letters* **2004**, *389* (1-3), 43-50.
(33) Kira, M.; Koch, S. W. *Semiconductor quantum optics*; Cambridge University Press, 2011.
(34) Kormányos, A.; Burkard, G.; Gmitra, M.; Fabian, J.; Zólyomi, V.; Drummond, N. D.; Fal'ko, V. k.p theory for two-dimensional transition metal dichalcogenide semiconductors. *2d Materials* **2015**, *2* (2).
(35) Laturia, A.; Van de Put, M. L.; Vandenberghe, W. G. Dielectric properties of hexagonal boron nitride and transition metal dichalcogenides: from monolayer to bulk. *Npj 2d Materials and Applications* **2018**, *2*.
(36) Malitson, I. H. Refraction and Dispersion of Synthetic Sapphire. *Journal of the Optical Society of America* **1962**, *52* (12), 1377-&.
